# Reinforcing one-carbon metabolism via folic acid/Folr1 promotes β-cell differentiation

**DOI:** 10.1038/s41467-021-23673-0

**Published:** 2021-06-07

**Authors:** Christos Karampelias, Habib Rezanejad, Mandy Rosko, Likun Duan, Jing Lu, Laura Pazzagli, Philippe Bertolino, Carolyn E. Cesta, Xiaojing Liu, Gregory S. Korbutt, Olov Andersson

**Affiliations:** 1 Department of Cell and Molecular Biology, Karolinska Institutet, Stockholm, Sweden; 2 Department of Surgery and Alberta Diabetes Institute, University of Alberta, Edmonton, Alberta Canada; 3 Department of Molecular and Structural Biochemistry, NC State University, Raleigh, NC USA; 4 Centre for Pharmacoepidemiology, Department of Medicine Solna, Karolinska Institutet, Stockholm, Sweden; 5 Cancer Research Centre of Lyon, INSERM U1052, CNRS UMR5286, Claude Bernard University, Lyon, France

**Keywords:** Differentiation, Regeneration, Type 1 diabetes

## Abstract

Diabetes can be caused by an insufficiency in β-cell mass. Here, we performed a genetic screen in a zebrafish model of β-cell loss to identify pathways promoting β-cell regeneration. We found that both folate receptor 1 (*folr1*) overexpression and treatment with folinic acid, stimulated β-cell differentiation in zebrafish. Treatment with folinic acid also stimulated β-cell differentiation in cultures of neonatal pig islets, showing that the effect could be translated to a mammalian system. In both zebrafish and neonatal pig islets, the increased β-cell differentiation originated from ductal cells. Mechanistically, comparative metabolomic analysis of zebrafish with/without β-cell ablation and with/without folinic acid treatment indicated β-cell regeneration could be attributed to changes in the pyrimidine, carnitine, and serine pathways. Overall, our results suggest evolutionarily conserved and previously unknown roles for folic acid and one-carbon metabolism in the generation of β-cells.

## Abstract (web summary)

Regeneration of insulin-producing beta-cells may become a future alternative treatment of diabetes. Here the authors report a genetic screen in a zebrafish model that mimics the loss of beta-cells in diabetes, and identified that the folate receptor Folr1 or folinic acid treatment can stimulate beta-cell regeneration.

## Introduction

Insulin-secreting pancreatic β-cells have a central role in regulating glucose homeostasis. Patients with type 1 or type 2 diabetes suffer from a loss of functional β-cells, manifesting as a central aspect of disease progression. In type 1 diabetes, β-cells are gradually depleted due to autoimmune attack^1^. Although type 2 diabetes arises from a different etiology, the later stages of the disease are characterized by a reduction in the functional β-cell mass due to apoptosis or dedifferentiation to a more immature stage^2,3^. The most successful current curative approach to tackle type 1 diabetes is islet/pancreas transplantation, which is hampered by a shortage of donors. Exogenous insulin administration and different drug regimens can treat the disease but cannot regulate the glucose levels as tightly as innate physiological regulation. Therefore, regeneration of the pancreatic β-cell population is an attractive future therapeutic alternative to the current treatments—one that could manage diabetes in a physiological manner.

β-cell regeneration in various preclinical animal models of diabetes has been shown to take place through three main mechanisms: the proliferation of preexisting β-cells, reprogramming of other pancreatic cell types to β-cells, and differentiation of duct-residing pancreatic progenitors to β-cells^4^. However, several caveats exist. The proliferation rate of adult human β-cells is very low, and the reprogramming of other pancreatic cell types to β-cells also occurs at a very low rate in vivo^5–8^. Differentiation of pancreatic progenitors that reside in or along the pancreatic duct has been proposed as an intriguing mechanism of β-cell regeneration, although there is controversy over whether cells with progenitor potential exist in the adult mammalian pancreas^9–16^.

The zebrafish is a popular model for studying tissue regeneration, as it can regenerate most of its tissues upon damage^17^. One zebrafish model that mimics the loss of β-cells in diabetes is based on a chemical ablation method using nitroreductase (NTR) and metronidazole (MTZ)^18,19^. Briefly, transgenic zebrafish express the enzyme NTR under the control of the insulin promoter, and upon administration of the prodrug MTZ, NTR converts MTZ to a toxic byproduct that specifically ablates the β-cell population. After β-cell ablation, the zebrafish has the ability to regenerate its β-cell population. Several chemical and genetic screens have uncovered signaling pathways potentiating β-cell differentiation and regeneration through different cellular mechanisms^20–26^. Interestingly, in the zebrafish model, duct-residing progenitors have been shown to exist and differentiate upon injury, making it an attractive model to study the biology of this progenitor population^27,28^.

Here, we performed a genetic screen to identify pathways that can promote β-cell regeneration in zebrafish. We characterized the transcriptome of regenerating islets from zebrafish and overexpressed the secreted proteins that were shown to be upregulated upon β-cell ablation. Overexpression of *folr1* stimulated β-cell regeneration by increasing duct-residing pancreatic progenitor differentiation. Folate receptors are responsible for the uptake of folic acid (also referred to as folate or vitamin B9) and its derivatives into cells and can be either secreted or bound to the cell membrane by a glycosylphosphatidylinositol (GPI) anchor. Treatment with folinic acid (known also as 5-formyl-THF or leucovorin) increased β-cell differentiation in both zebrafish and cultures of neonatal pig islets, suggesting that folic acid and one-carbon metabolism can potentiate β-cell differentiation across species. Folic acid is an important vitamin of the one-carbon metabolism pathway that provides carbon units for numerous cellular processes^29,30^, which are conserved from zebrafish to humans^31,32^. Given how tolerable folic acid is, our results encourage further exploration of its translational potential in preventing or managing diabetes.

## Results

### A genetic screen identifies *folr1* as an inducer of β-cell regeneration in zebrafish

**Fig. 1:**
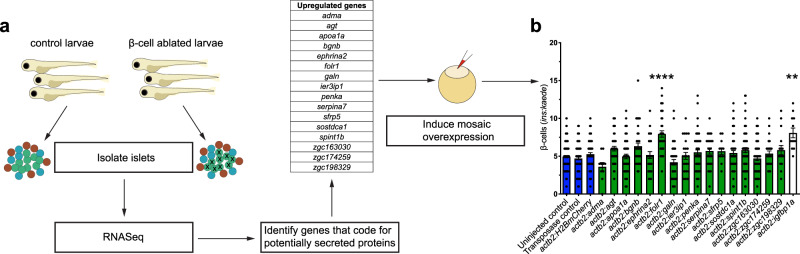
Genetic screen identifies *folr1* as an inducer of β-cell regeneration in zebrafish. **a** Schematic showing the experimental design of the genetic screen for inducers of β-cell regeneration. Briefly, primary islets were isolated from zebrafish larvae directly after β-cell ablation. RNA was extracted, and RNA-Seq was performed. Then, using the signalP algorithm, we identified the proteins with a signal peptide mediating secretion and cloned them under the control of the *actb2* promoter. The constructs were injected into 1-cell-stage embryos to induce mosaic overexpression, and the number of regenerating β-cells was quantified two days after β-cell ablation. **b** Results of the genetic screen for inducers of β-cell regeneration. Blue bars depict three different negative controls of the regeneration assay, green bars show the tested proteins and the white bar shows the regenerating β-cells for the positive control *igfbp1a*. Data for the negative controls were pooled from 4 independent experiments. If there was an observed increase in β-cell regeneration in the first experiment, constructs were retested a second time, and the data shown in the graph were pooled from both independent replicates. The Kruskal–Wallis test followed by Dunn’s multiple comparison test was performed. *n* = 137 (uninjected control), *n* = 108 (transposase control), *n* = 83 (*actb2:H2BmCherry*), *n* = 10 (*actb2:adma*), *n* = 42 (*actb2:agt*), *n* = 57 (*actb2:apoa1a*), *n* = 37 (*actb2:bgnb*), *n* = 26 (*actb2:ephrina2*), *n* = 38 (*actb2:folr1*), *n* = 39 (*actb2:galn*), *n* = 24 (*actb2:ier3ip1*), *n* = 58 (*actb2:penka*), *n* = 26 (*actb2:serpina7*), *n* = 12 (*actb2:sfrp5*), *n* = 54 (*actb2:sostdc1a*), *n* = 63 (*actb2:spint1b*), *n* = 62 (*actb2:zgc:163030*), *n* = 26 (*actb2:zgc:174259*), *n* = 16 (*actb2:zgc:198329*) and *n* = 12 (*actb2:igfbp1a*) biologically independent zebrafish larvae were used for the quantification of β-cells. ****P < 0.001, **P = 0.0061 compared to uninjected control. Data are presented as mean values ± SEM.

In this study, we ablated β-cells in zebrafish larvae using the NTR–MTZ system to identify pathways that are induced and can enhance β-cell regeneration. To this end, we characterized the transcriptome of isolated control islets and islets after β-cell ablation using RNA-Seq. By isolating the islets together with their surrounding acinar, ductal and endothelial cells, we aimed to identify pathways that act in the islet microenvironment and could stimulate β-cell regeneration. We focused our analysis on the upregulated genes that code for secreted proteins, i.e., proteins with a signal peptide targeting them for secretion, to enrich for genes affecting signaling pathways. We identified 16 upregulated genes that code for secreted proteins and cloned them downstream of a ubiquitous promoter (*actb2*). Subsequently, we injected these constructs into the zebrafish β-cell ablation model to induce mosaic overexpression of these genes and assessed their ability to enhance β-cell regeneration (Fig. 1a). Briefly, following injection of the constructs, we ablated the β-cells in transgenic zebrafish larvae expressing the fluorescent protein Kaede and NTR under the control of the insulin promoter, i.e., *Tg(ins:kaede);Tg(ins:CFP-NTR)*. We administered MTZ between 3 and 4 days postfertilization (dpf) to ablate the β-cells and then allowed regeneration to proceed for 2 days (4–6 dpf), a procedure we refer to as the β-cell regeneration assay. Our genetic screen revealed that *folr1* overexpression could stimulate β-cell regeneration at supraphysiological levels, and the increase was on par with that in our positive control *igfbp1a* (Fig. 1b).

**Fig. 2:**
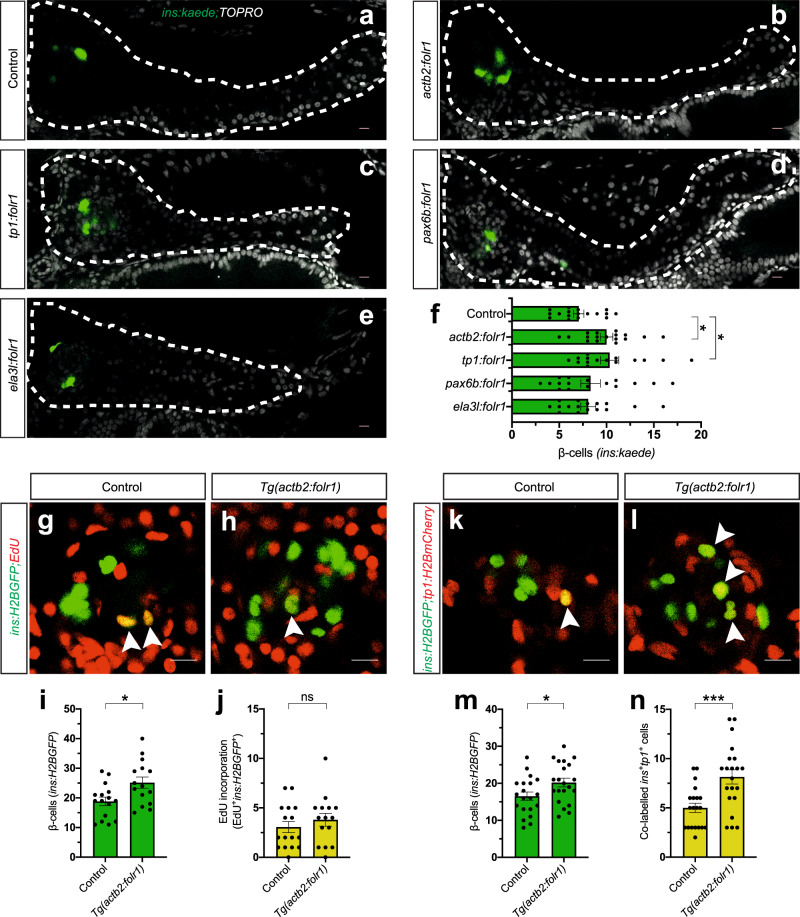
*folr1* overexpression increases β-cell regeneration from a ductal source. **a**–**f** Single-plane confocal pictures of pancreata in control (**a**), *actb2:folr1* (**b**), *tp1:folr1* (**c**), *pax6b:folr1* (**d**), and *ela3l:folr1* (**e**) larvae on the *Tg(ins:kaede);Tg(ins:CFP-NTR)* background, following two days of β-cell regeneration. TOPRO was used to counterstain nuclei, and the whole pancreas is outlined with a white dashed line. Quantification of the pancreatic β-cells showed that *actb2:folr1* and *tp1:folr1* overexpression had comparable increases in the β-cell regeneration assay (**f**). Scale bar, 10 μm. *n* = 17 (control), *n* = 17 (*actb2:folr1*), *n* = 15 (*tp1:folr1*), *n* = 16 (*pax6b:folr1*), and *n* = 16 (*ela3l:folr1*) biologically independent zebrafish larvae used for the quantification of β-cells. Data are presented as mean values ± SEM. One-way ANOVA was performed followed by Dunnett’s multiple comparison tests. **f** *P = 0.0348 (control vs. *actb2:folr1*), *P = 0.0202 (control vs *tp1:folr1*). **g–j** Single-plane confocal images of islets in control (**g**) and *Tg(actb2:folr1)* (**h**) larvae on the *Tg(ins:H2BGFP);Tg(ins:flag-NTR)* background following two days of β-cell regeneration while incubated with EdU to label proliferating cells. Quantification showed an increase in β-cell regeneration in the *Tg(actb2:folr1)* line (**i**), but no change in the number of β-cells incorporating EdU was observed (**j**). Arrowheads point to EdU^+^ins:H2BGFP^+^ cells. Scale bar, 10 μm. *n* = 16 control and *n* = 15 *Tg(actb2:folr1)* biologically independent zebrafish larvae were used for the quantification of this experiment. Data are presented as mean values ± SEM. Unpaired two-tailed Student’s *t* test was used to assess significance. **i** *P = 0.0122, **j** nonsignificant (ns), *P* = 0.3943. **k**–**n** Single-plane confocal images of islets in control (**k**) and *Tg(actb2:folr1)* (**l**) larvae on the *Tg(ins:H2BGFP);Tg(ins:flag-NTR);Tg(tp1:H2BmCherry)* background, used to lineage trace the ductal cells of the pancreas, after β-cell ablation. Quantification showed an increase in the number of regenerating β-cells (**m**) colabeled with the ductal cell marker *tp1:H2BmCherry* (**n**). Arrowheads point to tp1:H2BmCherry^+^ins:H2BGFP^+^ cells. Data for **m**, **n** were pooled from two independent experiments. Scale bar, 10 μm. *n* = 20 control and *n* = 21 *Tg(actb2:folr1)* biologically independent zebrafish larvae were used for the quantification of samples pooled from two independent experiments. Data are presented as mean values ± SEM. Unpaired two-tailed Student’s *t* test was used to assess significance for (**m**) *P = 0.0306. The Mann–Whitney two-tailed test was used for (**n**) ***P = 0.0010.

Folate receptors can either reside on the cell membrane by a GPI anchor or shed off to act in a non-cell-autonomous fashion. We cloned *folr1* under the control of different tissue-specific promoters to assess whether the increase in β-cell regeneration upon *folr1* overexpression is mediated cell-autonomously within a certain tissue or cell type. We used the promoters of *pax6b* (expressed in endocrine cells), *tp1* (i.e., a Notch-responsive element from the Epstein Barr virus, expressed in the pancreatic duct), and *ela3l* (expressed in acinar cells) to overexpress *folr1* in various cell types of the pancreas. Ductal-restricted overexpression was the only cell type-specific overexpression of *folr1* that increased β-cell regeneration to similar levels as ubiquitous overexpression (Fig. 2a–f). To strengthen this conclusion, we generated two additional *folr1* constructs. The first construct lacked the predicted GPI anchor domain responsible for attaching it to the membrane. The second construct lacked the predicted signal peptide that targets Folr1 for secretion, as well as we attached a nuclear localization signal (NLS) to the C-terminus. The reason to generate the *folr1-NLS* construct is that Folr1 has been postulated to act as a transcription factor, under certain in vitro conditions^33^. Therefore, we wanted to assess the possibility that our observed phenotype stems from Folr1 acting as a transcription factor by reinforcing its localization to the nucleus. We overexpressed the constructs in the ductal cells using the *tp1* promoter. Zebrafish that overexpressed either truncated *tp1:folr1* lacking the GPI anchor binding domain or the *tp1:folr1-NLS* construct had lost the ability to stimulate β-cell regeneration, indicating that attachment of Folr1 to the membrane of the ductal cells is important for its effect (Supplementary Fig. 1) and that Folr1 does not act as a transcription factor in this context. These results suggest that membrane-attached *folr1* overexpression acts autonomously in the pancreatic duct to stimulate β-cell regeneration.

We then expanded our investigation on the cellular mechanism of β-cell regeneration upon *folr1* overexpression. We generated a transgenic zebrafish line stably overexpressing *folr1* under the control of the *actb2* promoter (referred to as *Tg(actb2:folr1)*). We focused our studies on the single primary islet of zebrafish larvae, as we did not observe the precocious formation of secondary islets along the tail of the pancreas at this stage. We assessed the β-cell proliferative capacity in the *Tg(actb2:folr1)* larvae by examining the incorporation of the thymidine analog EdU in the β-cell regeneration assay. We did not observe a difference in β-cell proliferation between control and *Tg(actb2:folr1)* larvae (Fig. 2g–j). Next, we lineage-traced the ductal cells short-term using *Tg(tp1:H2BmCherry)* in the β-cell regeneration assay. In this transgenic zebrafish line, the fluorescent protein H2BmCherry stably marks ductal cells. *Tg(actb2:folr1)* larvae showed an increase in β-cells co-labeled with the ductal cell marker H2BmCherry (Fig. 2k–n). Together, our genetic screen revealed a previously unknown role for *folr1* in promoting β-cell regeneration from a ductal source in zebrafish larvae.

### Expression of Folr1 in the pancreata of different organisms

**Fig. 3:**
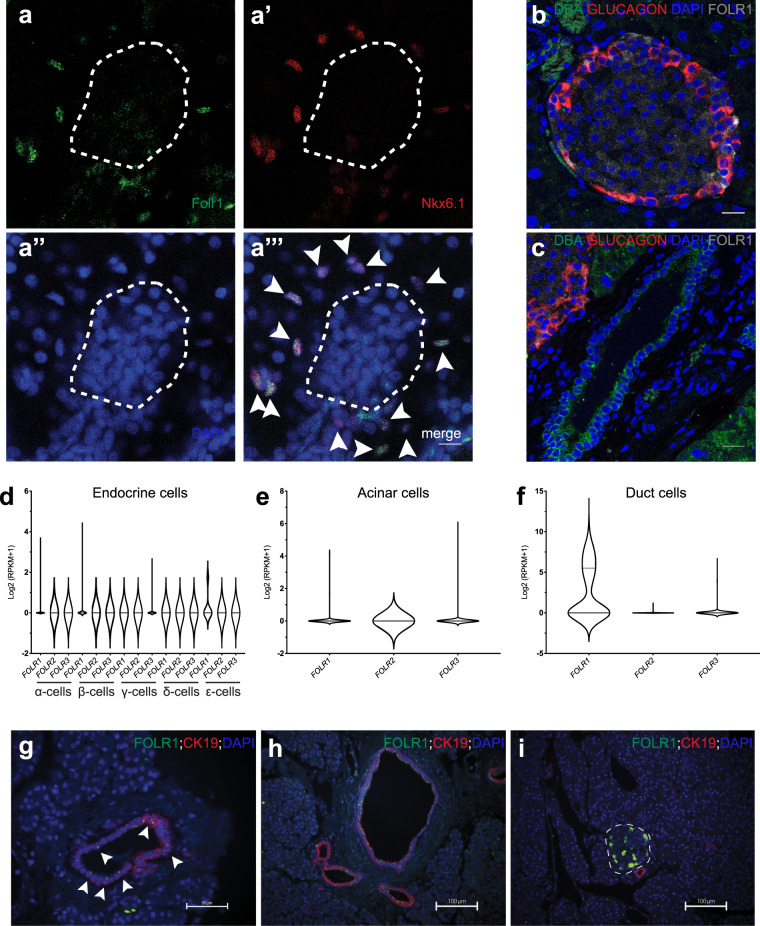
Expression of Folr1 in the pancreata of different organisms. **a**–**a**”’, Immunostaining of whole-mount zebrafish larvae at 6 dpf using a Folr1 antibody (**a**). Ductal cells were immunostained with the zebrafish duct-specific marker Nkx6.1 (**a**’), and nuclei were counterstained with DAPI (**a**”). The white dashed line outlines the islet of the zebrafish larvae. Arrowheads (**a**”’) point to the Folr1^+^ ductal cells. Scale bar, 10 μm. Representative images are shown from one larva. The staining results have been repeated in at least four zebrafish larvae and three biological replicates. **b**, **c** Representative pictures of an islet (**b**) and a duct (**c**) from adult mouse pancreatic sections immunostained for FOLR1, GLUCAGON (α-cell marker), and DBA (ductal cell marker) and counterstained with DAPI. Scale bar, 20 μm. Sections from three biological replicates showed the same expression pattern of FOLR1. **d**–**i** Expression of folate receptor homologs in the human pancreas. Violin plots showing single-cell RNA-Seq expression in endocrine (**d**), acinar (**e**), and ductal (**f**) pancreatic cells showed significant expression of *FOLR1* in a subset of duct cells. The expression pattern of *FOLR1* found in the single-cell RNA-Seq data was confirmed with immunofluorescence analysis of human pancreatic sections (**g**–**i**). The white dashed line outlines the islet in (**i**). Arrowheads in **g** point to the subset of ductal cells expressing FOLR1, whereas essentially all ductal cells in the large duct in (**h**) are FOLR1+. Scale bars are indicated on the images. The staining pattern was consistent in pancreata from eight different donors.

As overexpression of *folr1* in ductal cells increased β-cell regeneration, we wondered what endogenous expression pattern of Folr1 appeared in the pancreata of different organisms. In the zebrafish larvae, we found Folr1 to be expressed in ductal cells and the islet (Fig. 3a–a”’). In contrast, adult mice expressed FOLR1 in only the islets of pancreata with no expression in ductal or acinar cells (Fig. 3b, c). When we explored the expression of *FOLR1* in previously published human single-cell RNA-Seq data, we found that it was restricted to a subset of human ductal cells, with modest expression in other tissues^34,35^. Notably, none of the other folate receptor homologs were found to be expressed in the pancreas at high levels (Fig. 3d–f). Immunostaining of human pancreatic sections against FOLR1 showed a speckle-like staining pattern within ductal cells, indicating poor localization of FOLR1 to the plasma membrane (Fig. 3g, h). FOLR1 immunoreactivity was observed in a subset of ductal cells in certain ducts while in all ductal cells in other ducts (Fig. 3g, h), highlighting expression heterogeneity. We also observed FOLR1 expression in human islet cells (in contrast to the single-cell RNA-Seq data) with cytoplasmic/plasma membrane localization (Fig. 3i). We then took advantage of one of the published single-cell RNA-Seq datasets of the human pancreas^35^ and examined whether the expression of any mRNA correlated with *FOLR1* in ductal cells. Although we did not observe strong positive or negative correlations with *FOLR1* (rho > ±0.8), we observed that there were a few genes that correlated weakly but significantly with *FOLR1* (rho > 0.2) (Supplementary Fig. 2). Interestingly, one of the top genes was *CEACAM6*, which has been proposed to be a marker of human pancreatic progenitors using the StemID algorithm^36^. Together, Folr1 is expressed in a subset of pancreatic ductal cells in zebrafish and humans but not in mice.

### One-carbon metabolism stimulates β-cell regeneration

**Fig. 4:**
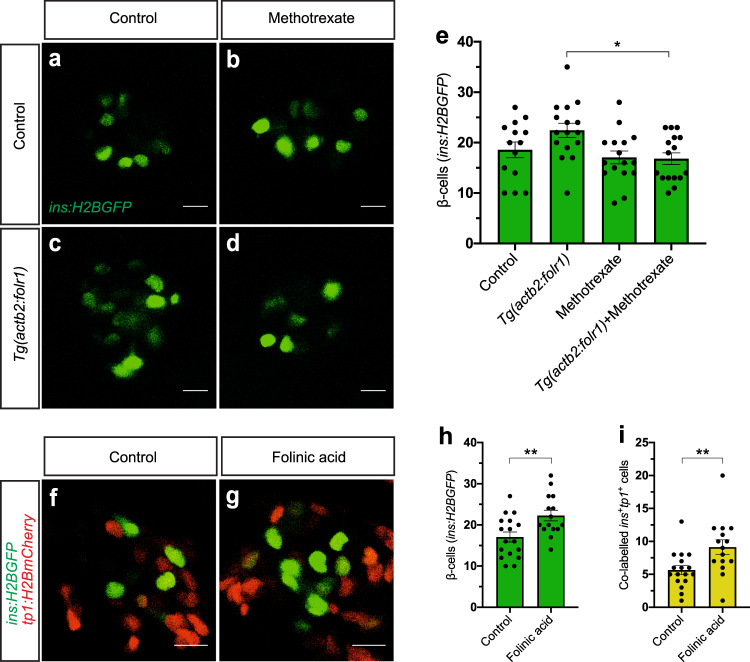
One-carbon metabolism stimulates β-cell regeneration. **a**–**e** Single-plane confocal images of control (**a**), methotrexate-treated (**b**), *Tg(actb2:folr1)* (**c**) and *Tg(actb2:folr1)* cotreated with methotrexate (**d**) islets following 2 days of β-cell regeneration. Quantification showed that the number of regenerating β-cells upon *Tg(actb2:folr1)* overexpression was reduced to the baseline level after treatment with methotrexate (**e**). Scale bar, 10 μm. *n* = 14 control, *n* = 16 methotrexate-treated, *n* = 16 *Tg(actb2:folr1)*, and *n* = 16 *Tg(actb2:folr1)* cotreated with methotrexate biologically independent zebrafish larvae were used for the quantification of β-cells. Data are presented as mean values ± SEM. One-way ANOVA was used to assess significance followed by Holm–Sidak’s multiple comparison test. *P = 0.0120. **f**–**i** Folinic acid treatment for two days following β-cell ablation increased β-cell regeneration from ductal cells by 6 dpf in zebrafish larvae. Single-plane confocal images of *Tg(ins:H2BGFP)*;*Tg(ins:flag-NTR)*;*Tg(tp1:H2BmCherry)* control (**f**) and folinic acid-treated (**g**) larvae, along with the quantification of the β-cells (**h**) and the β-cells co-labeled with the ductal cell tracer *tp1:H2BmCherry* (**i**). Scale bar, 10 μm. *n* = 17 (control) and *n* = 15 (folinic acid) biologically independent zebrafish larvae were used for the quantification of this experiment. Data are presented as mean values ± SEM. Unpaired two-tailed Student’s *t* test was used for (**h**) **P = 0.0064. Unpaired two-tailed Student’s *t*-test was used to assess significance for (**i**) **P = 0.0094.

Folr1 binds and internalizes folic acid and its derivatives into cells to be used for a variety of biochemical reactions. Therefore, we decided to further explore the involvement of one-carbon metabolism in β-cell regeneration. First, we treated *Tg(actb2:folr1)* zebrafish larvae with methotrexate in the β-cell regeneration assay. Methotrexate blocks the synthesis of tetrahydrofolate (THF), which is the metabolically active form of folic acid in cells and a precursor of the major circulating form of folic acid (5-methyl-THF). Inhibition of THF biosynthesis abolished the effect of *Tg(actb2:folr1)* on β-cell regeneration (Fig. 4a–e). Second, we treated zebrafish larvae with the stable folate intermediate, folinic acid, following β-cell ablation and observed an increase in β-cell regeneration (Supplementary Fig. 3), albeit not as potently as during overexpression of *folr1*. Moreover, folinic acid treatment increased the number of β-cells co-labeled with the ductal tracer *tp1:H2BmCherry*, similarly to our *Tg(actb2:folr1)* genetic model (Fig. 4f–i). These data suggest that reinforcing the one-carbon metabolism can induce β-cell regeneration and mediates the effect of *folr1* overexpression in zebrafish larvae.

### One-carbon metabolism activation does not alter the β-cell number in the basal state

As reinforcing one-carbon metabolism increased β-cell regeneration in the zebrafish larvae, we also sought to analyze the effect on the number of β-cells in the basal state, i.e., in zebrafish larvae under physiological conditions. We used both the *Tg(actb2:folr1)* larvae as well as larvae treated with folinic acid for 48 hours, in the *Tg(ins:H2BGFP)* background to clearly visualize the number of β-cells. We observed that neither way of reinforcing one-carbon metabolism increased the number of β-cells in the basal state, suggesting that the increase in β-cell number is restricted to β-cell regeneration (Supplementary Fig. 4). Since the newly formed β-cells were derived from a ductal origin, we asked whether folinic acid treatment affected the number or proliferation status of the ductal cells. We cotreated *Tg(ins:flag-NTR);Tg(tp1:GFP)* larvae with folinic acid and EdU during the regenerative period and found that the number and proliferation rate of ductal cells were unaffected by the treatment (Supplementary Fig. 5a–d). These results indicate that the increase in β-cell regeneration, through ductal to β-cell differentiation, was not an indirect phenotype caused by a larger pool of ductal cells.

### Folinic acid stimulates δ-cell regeneration

Our data suggested that reinforcing one-carbon metabolism directly affects ductal cells to differentiate into β-cells after β-cell ablation. Therefore, we reasoned that folinic acid might also stimulate the differentiation of ductal cells to other endocrine cell types after reciprocal ablation. To answer this question, we ablated somatostatin-producing δ-cells using *Tg(sst2:NTR);Tg(sst2:dsRed2)* zebrafish larvae and subsequently treated them with folinic acid for 48 hours, similar to the method used in our β-cell regeneration assay. Consistent with our hypothesis, we noted an increase in regenerating δ-cells after treatment with folinic acid (Supplementary Fig. 5e–g). Thus, these data suggest that the regeneration induced by one-carbon metabolism is not restricted to β-cells but is also applicable to at least one more endocrine cell type, the δ-cell.

### Folinic acid also increases β-cell regeneration in 1-month-old zebrafish

**Fig. 5:**
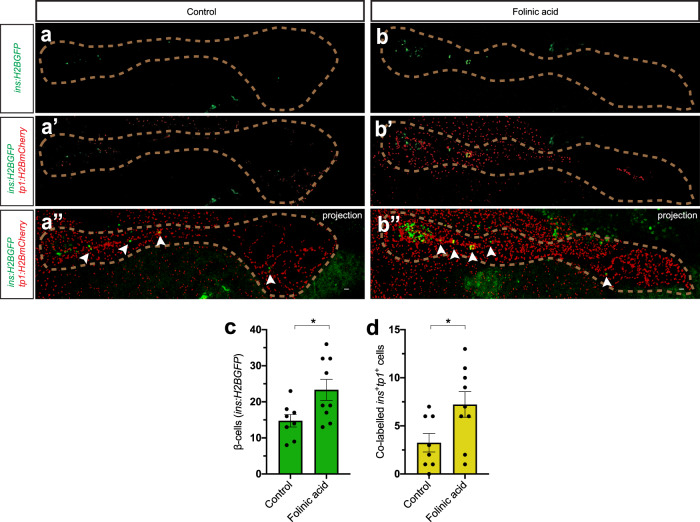
Folinic acid stimulates β-cell regeneration in juvenile zebrafish. **a**–**d** Single-plane and maximum projection confocal images of pancreata in control (**a**–**a**”) or folinic acid-treated (**b**–**b**”) 1-month-old juvenile zebrafish on the *Tg(ins:H2BGFP)*;*Tg(ins:flag-NTR)*;*Tg(tp1:H2BmCherry)* background, following β-cell ablation. Quantification showed an increase in the number of regenerating β-cells in the secondary islets upon treatment with folinic acid (**c**) as well as an increase in the number of *ins:H2BGFP*^+^*tp1:H2BmCherry*^*+*^ cells (**d**). The dashed line outlines the pancreas. Arrowheads point to ins:H2BGFP^+^ cells in the secondary islets. Scale bar, 20 μm. *n* = 8 (control) and *n* = 9 (folinic acid) biologically independent zebrafish were used for the quantification of this experiment. Data are presented as mean values ± SEM. Unpaired two-tailed Student’s *t* test was used to assess significance for (**c**) and (**d**). **c** *P = 0.0264; **d** *P = 0.0319.

Our results suggest that reinforcing one-carbon metabolism can increase β-cell regeneration in zebrafish larvae. To test whether this effect is conserved in older zebrafish, we ablated the β-cells in 1-month-old *Tg(ins:flag-NTR);Tg(ins:H2BGFP);Tg(tp1:H2BmCherry)* zebrafish and subsequently treated the zebrafish with folinic acid for 2 days. We quantified the number of regenerated β-cells in the secondary islets and how many of them colocalized with the ductal cell tracer *tp1:H2BmCherry*. Treatment with folinic acid increased the total number of regenerating β-cells in the secondary islets and the number of β-cells derived from the ductal cell population (Fig. 5). Together, these results demonstrate that the effect of folinic acid treatments in larvae is conserved in older zebrafish.

### Metabolomics reveals regulation of pyrimidine, carnitine, and serine metabolic pathways in response to folinic acid and β-cell regeneration

**Fig. 6:**
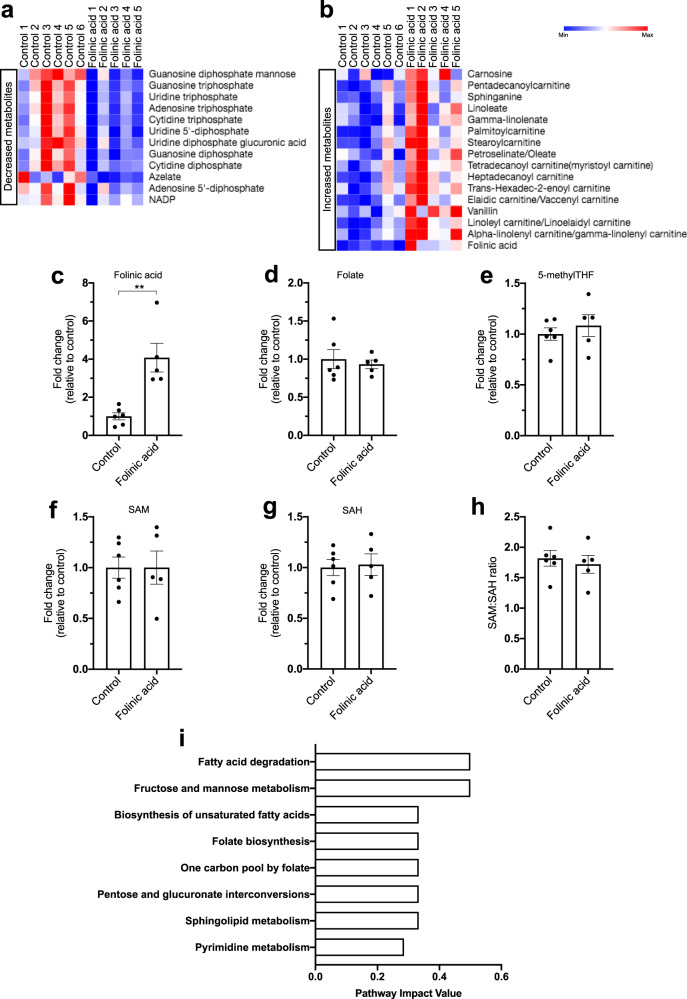
Metabolomics characterization of folinic acid-treated zebrafish larvae following β-cell ablation. **a**, **b** Heat-maps showing the significantly decreased (**a**) and increased (**b**) metabolites upon folinic acid treatment for 24 h, following β-cell ablation in the *Tg(ins:flag-NTR)* line. **c**–**e** Changes in the level of folinic acid (**c**), folate (**d**), and 5-methylTHF (**e**) reported as fold change to the untreated zebrafish larvae after β-cell ablation following 24 h of folinic acid treatment. *n* = 6 (control) and *n* = 5 (folinic acid) biologically independent metabolite preparations from five pooled larvae each were used for the quantification of this experiment. Data are presented as mean values ± SEM. Mann–Whitney two-tailed test was used to assess statistical significance. **c** **P = 0.0043. **f**–**h** Folinic acid treatment does not affect the levels of SAM (**f**), SAH (**g**), or their ratio (**h**). *n* = 6 (control) and *n* = 5 (folinic acid) biologically independent metabolite preparations from five pooled larvae each were used for the quantification of this experiment. Data are presented as mean values ± SEM. **i** Pathway impact values of zebrafish-specific enriched pathways for the significantly affected metabolites (Fig. 6a, b).

To further investigate how folinic acid treatment might affect one-carbon metabolism and stimulate β-cell regeneration, we performed metabolomic profiling of zebrafish larvae. To this end, we extracted polar metabolites from 5 dpf zebrafish larvae with/without β-cell ablation as well as with/without folinic acid treatment. Then, we compared the different datasets to assess the metabolites with significantly altered levels. Folinic acid treatment without β-cell ablation, i.e., in the basal state, had a modest effect on the metabolome of the zebrafish. On the contrary, folinic acid treatment significantly changed the level of a number of metabolites, following β-cell ablation (Fig. 6a, b). Specifically, the number of nucleotides was significantly decreased upon folinic acid treatment (Fig. 6a), whereas we observed a strong increase in the levels of certain fatty acids and carnitine-related metabolites (Fig. 6b). Next, we quantified the levels of different folate cycle intermediates, to assess changes in the folate cycle under these conditions. We observed a strong increase in the folinic acid level, as expected, but no changes in the folate or 5-methyl-THF levels (Fig. 6c–e). 5-methyl-THF is the predominant folate intermediate that provides the carbon to generate methionine from homocysteine and thus affects methylation reactions. Therefore, we also examined the levels of s-adenosyl methionine (SAM), which is derived from methionine and is considered the methyl donor for methylation reactions, s-adenosyl homocysteine (SAH), the metabolic product of the methylation reaction, as well as their ratio. We did not observe any shift in the SAM and SAH levels or their ratio, which is thought to reflect the methylation status^37^ (Fig. 6f–h). To identify the metabolic pathways that might be responsible for the observed increase in β-cell regeneration following folinic acid treatment, we performed a zebrafish-specific pathway enrichment analysis for the significantly altered metabolites. Similar to our initial observations, pathway analysis confirmed that metabolites with significantly altered levels were enriched for the biosynthesis and degradation of unsaturated fatty acids as well as sphingolipid metabolism, showing that folinic acid treatment affects fatty acid metabolism (Fig. 6i). Moreover, our pathway enrichment analysis demonstrated that folinic acid treatment affected the folate biosynthesis and one carbon pool by folate pathways in zebrafish, as well as metabolites in the pyrimidine metabolic pathway (Fig. 6i). Thus, using a metabolomics approach our data implicate pyrimidine and fatty acid-related metabolic processes in the folinic acid-induced β-cell regeneration in vivo.

Zebrafish have a remarkable β-cell regenerative capacity. In order to compare how folinic acid treatment could accelerate this process, we asked what were the metabolites that significantly changed in the untreated conditions, i.e., from the basal state to the regeneration state. To that end, we compared metabolites from zebrafish with and without β-cell ablation, similarly to our initial genetic screen (Fig. 1). A great number of metabolites were significantly affected by β-cell ablation (Supplementary Data 1). Zebrafish-specific pathway analysis for metabolites with both increased and decreased levels revealed the glycine, serine and threonine metabolism as the most impacted pathway with multiple metabolites significantly affected (8 out of 11 detected metabolites of the pathway were significantly altered) (Supplementary Fig. 6a, b). Interestingly, choline, serine and sarcosine are directly or indirectly involved in the donation of one-carbon units to folate species. Therefore, we investigated whether folinic acid treatment could affect the levels of these metabolites in our zebrafish model. Indeed, we observed that folinic acid treatment can partially reverse the level of four out of the eight metabolites of the glycine, serine, and threonine pathway, albeit not reaching statistical significance (Supplementary Fig. 6c–f). Therefore, our data could indicate a link between critical one-carbon metabolite donors to folates that could affect β-cell regeneration.

Our metabolomics analysis also indicated that folinic acid treatment can upregulate a number of carnitine-related metabolites during β-cell regeneration. When we investigated the levels of these exact metabolites in the untreated β-cell regeneration state compared to the basal state, i.e., neither group had folinic acid treatment, we noticed that the levels of the same carnitine-associated metabolites were significantly decreased (Supplementary Fig. 6g). Serine starvation has previously been postulated to have a role in carnitine and fatty acid metabolism^38^. Linking to our dataset, one of the metabolites that is decreased in the basal regenerative state but are increased upon folinic acid treatment is sphinganine, which is a crucial metabolite in the metabolic pathway that uses serine to synthesize ceramides (see “Discussion”). Overall, our metabolomics analysis reveals a potentially important regulation of serine-related and carnitine-linked metabolites in β-cell regeneration and following folinic acid treatment.

### Folinic acid promotes β-cell differentiation in an in vitro culture of neonatal pig islets

**Fig. 7:**
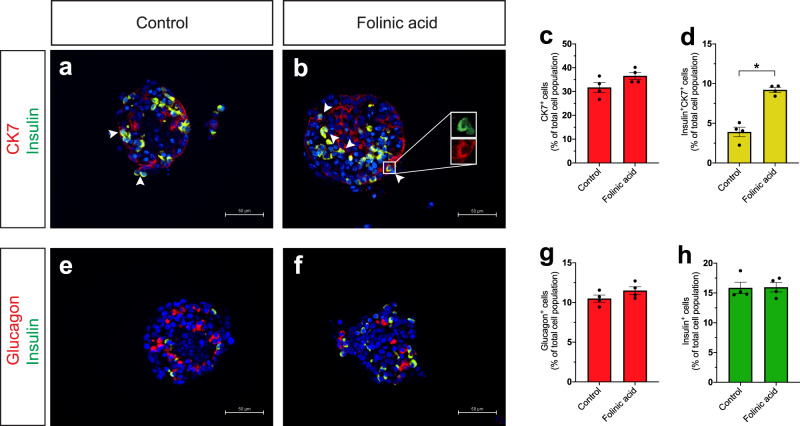
Folinic acid stimulates β-cell differentiation in neonatal pig islets. **a**–**d** Folinic acid stimulates β-cell differentiation from ductal cells in neonatal pig islets. Images of control (**a**) and folinic acid-treated (**b**) neonatal pig islets immunostained for insulin and CK7. Quantification of the percentage of CK7^+^ and CK7^+^Insulin^+^ cells is shown in (**c**) and (**d**), respectively. Arrowheads point to CK7^+^Insulin^+^ cells. *n* = 4 biologically independent pig islet preparations. Data are presented as mean values ± SEM. **d** *P = 0.0286. **e**–**h** Folinic acid did not increase the percentage of α- or β-cells in the in vitro neonatal pig islet culture. Control (**e**) and folinic acid-treated (**f**) neonatal pig islets immunostained for insulin and glucagon. Quantification of the percentage of α- and β-cells is shown in (**g**) and (**h**), respectively. *n* = 4 biologically independent pig islet preparations. Data are presented as mean values ± SEM. The scale bar is 50 μm for all pictures in the figure. All statistical analyses were performed with a Mann–Whitney two-tailed test.

To assess whether the effect of folinic acid on β-cell generation can be translated to a mammalian model, we used an in vitro culture system of neonatal pig islets. This model is ideal for studying the formation of β-cells from ductal cells because these cultures are rich in intraislet ducts. We started by examining if folinic acid could induce β-cell formation from ductal cells in neonatal pig islet cultures. To this end, we immunostained neonatal pig islets for CK7, a marker of ductal cells in pigs. We did not observe a significant difference in the percentage of ductal cells after folinic acid treatment, but the treatment increased the percentage of CK7^+^INS^+^ cells in the pig islet cultures (Fig. 7a–d). However, this did not translate to an increased percentage of β- or α-cells in the pig islet cultures (Fig. 7e–h). Nevertheless, somatostatin^*+*^ cells were significantly increased upon folinic acid treatment (Supplementary Fig. 7a-c), correlating with the increased δ-cell regeneration we observed in the zebrafish larvae. In addition, we quantified the glucose-stimulated insulin secretion and found that the β-cells were equally functional after folinic acid treatment as they were after the control treatment (Supplementary Fig. 7d). The data suggested that folinic acid can stimulate the process of β-cell neogenesis from ductal cells but perhaps needs a longer treatment window to produce fully mature β-cells. Together, these results indicate that the effect of folinic acid on endocrine cell differentiation from ductal cells is conserved from fish to mammals.

## Discussion

In this study, we performed an unbiased genetic screen to identify inducers of pancreatic β-cell regeneration. By using RNA-Seq characterization of the regenerating islet environment after β-cell injury, we identified and tested 16 upregulated proteins with signal peptide-mediated secretion. One of them, Folr1, uncovered a previously unknown role for one-carbon metabolism in stimulating β-cell differentiation in the zebrafish and pig models. Three cellular mechanisms of β-cell regeneration, i.e., the proliferation of preexisting β-cells, reprogramming other cell types to β-cells, as well as neogenesis from progenitors residing in the duct of the pancreas, have been shown to contribute to the newly formed β-cells in zebrafish larvae^39^. *folr1* overexpression specifically in the ductal cell compartment and short-term lineage-tracing experiments suggest that the newly formed β-cells most likely were derived from ductal cells. However, an inducible lineage-tracing approach is needed to conclusively claim that the source of the regenerating β-cells upon activation of one-carbon metabolism is ductal cells.

Folinic acid treatments increased β-cell differentiation in zebrafish and in vitro in our neonatal porcine islet culture. The increase in insulin colocalizing with the ductal cell marker CK7, further strengthening our observations in zebrafish that β-cells can derive from ductal residing progenitors. As we mentioned previously, the progeny of these embryonic ductal cells have been shown to contribute to β-cell regeneration in adult zebrafish^27,28^. However, the existence of similar progenitors in mammalian models is still under debate. Genetic lineage tracing in mice, with proposed markers of this population, including Sox9, Hes1 and Hnf1b, has failed to detect differentiation of ductal cells to endocrine cells in the adult mouse^9–11^. On the other hand, a recent study proposed that Aldh1b1^+^ centroacinar cells can contribute to all pancreatic lineages, corroborating previous research in mice and zebrafish regarding the differentiation potential of pancreatic progenitors^40–42^. Moreover, certain experimental procedures that are used to study β-cell regeneration, such as pancreatic duct ligation, seem to reawaken an embryonic progenitor signature in mammalian ductal cells, and in some circumstances, these cells have been shown to contribute to β-cell formation^13,15,43,44^. Our in vitro cultures of neonatal porcine islets include abundant amounts of intraislet ductal cells. Interestingly, intraislet ducts are also present in young mice and can contribute to β-cell formation^45^. Whether these intraislet ductal cells contain a subpopulation of cells that are capable of differentiating to endocrine cells upon injury, as well as for how long they or similar cells remain in the pancreas, should be further explored.

Analysis of published single-cell RNA-Seq data of human pancreata showed that *FOLR1* is expressed specifically in a subset of ductal cells. However, immunofluorescence staining of endogenous FOLR1 was localized to speckles within the ductal cells, and perhaps overexpression or other means of localizing FOLR1 to the cell membrane is needed to increase cellular uptake of folate. In one of the datasets, we also observed a modest but significant correlation with CEACAM6. CEACAM6 has been proposed as a marker of human ductal progenitors based on the StemID algorithm^36^. Moreover, a study aimed to identify cell surface markers of pancreatic progenitors during human embryonic stem cell differentiation to β-cells pointed to FOLR1 as one of the top candidates^46^. Our study suggests that apart from being a marker of pancreatic progenitors, FOLR1 can have a functional role in differentiation to endocrine cells, including β- and δ-cells.

In this study, we performed a metabolomics analysis, to characterize the effect of folinic acid during β-cell regeneration. Using this approach, we also cataloged the metabolites that are significantly altered after β-cell ablation in zebrafish, providing a useful resource for future studies. The folate cycle is known to affect a number of important metabolic pathways in the cell, including methylation reactions and nucleotide metabolism^30^. Interestingly, our data indicate that the accelerated β-cell regeneration phenotype we observe is independent of the methionine cycle but seems to involve altered nucleotide metabolism. More importantly, the decrease in carnitine-linked metabolites during β-cell regeneration was partially reversed by folinic acid, when compared to the untreated controls. Serine metabolism has been shown to have a role in regulating carnitine-linked metabolites and ceramide synthesis to directly affect mitochondrial function^38,47^. The folinic acid-induced increase of carnitine-linked metabolites together with a decrease of ATP production indicating a reduction in β-oxidation of fatty acids can affect β-cell regeneration.

Overall, given that folic acid is safe and widely used, together with our encouraging results on β-cell generation in model systems, makes it an appealing supplement to be further studied during β-cell development and in diabetes.

## Methods

### Zebrafish experimental procedures and transgenic lines

Zebrafish experiments were conducted in compliance with local guidelines and approved by Stockholms djurförsöksetiska nämnd. The previously generated transgenic lines used include *Tg(ins:flag-NTR)*^*s950*^, *Tg(tp1:H2BmCherry)*^*s939*^, *Tg(tp1:GFP)*^*um14*^, *Tg(ins:CFP-NTR)*^*s892*^, *Tg(ins:Kaede)*^*s949*^*, Tg(ins:H2BGFP)*^*KI112*^, *Tg(sst2:NTR,cryaa:Cerulean)*^*KI102*^ and *Tg(sst2:dsRed2)*^*gz19*^.

In this work, we generated a stable line overexpressing *folr1* under the control of the *actb2* promoter, i.e., *Tg(actb2:folr1,myl7:EGFP)*^*KI115*^, whose overexpression was confirmed by qPCR on mRNA from whole larvae.

Chemical treatment of zebrafish larvae was performed by adding the chemicals to E3 buffer for 48 h unless otherwise stated. The concentrations of the chemicals used were 20 μM folinic acid (Sigma-Aldrich) and 10 μM methotrexate (Sigma-Aldrich).

Ablation of β-cells or δ-cells in the zebrafish larvae using *Tg(ins:flag-NTR)*, *Tg(ins:CFP-NTR)* or *Tg(sst2:NTR)* was performed by incubating the larvae for 24 hours with 10 mM metronidazole (Sigma-Aldrich) diluted in 1% DMSO (VWR) in E3 solution supplemented with 0.2 mM 1‐phenyl‐2‐thiourea (PTU, Acros Organics). For the juvenile stage, β-cell ablation was performed by incubating the zebrafish with 5 mM MTZ for 24 h, followed by treatment with folinic acid for 2 days while feeding.

### RNA-Seq

Islet isolation from zebrafish larvae and RNA extraction were described previously^23^. RNA-Seq and mapping to the Zv9 zebrafish genome assembly was performed as previously described using the Bowtie algorithm for filtering and TopHat algorithm for the alignment^48,49^. Data were normalized as fragments per kilobase of million mapped reads values, the fold change was calculated by dividing the normalized value of the control to the normalized value of the ablated condition and the data were managed using the LabxDB tool^50^. No statistical testing was performed to filter the data. All the genes with a fold change >2 were screened for a signal peptide targeting them for secretion using SignalP software (http://www.cbs.dtu.dk/services/SignalP/)^51^.

### Cloning of selected genes and genetic screening

To clone the selected upregulated genes, we amplified the gene sequences from pooled cDNA of different stages (3 hpf, 8 hpf, 1 dpf, 2 dpf, 3 dpf, 4 dpf, 5 dpf, and 6 dpf). Then, we cloned the genes into the middle donor vector of the Gateway system, i.e., pDONR221. Subsequently, we performed a three-way LR recombination reaction to create the final vector, where we recombined the 5′ entry vector containing the promoter sequences of choice, the middle entry vector containing the gene of interest, and the 3’ entry vector containing a polyA sequence into the destination vector pDESTtol2CG2, which carried a selection marker (*cmlc2:GFP)* to express GFP in cardiomyocytes. We succeeded in cloning 16 out of an initial pool of 22 genes into the final vector. Primers for the cloned genes are shown in Supplementary Table 1. Initially, 20 pg of the final vectors were injected into the 1-cell stage embryo together with 20 pg of transposase mRNA. If the overexpression caused toxicity in the first injection experiment, 10 pg of the vector was injected in the following experiments. Larvae that had integrated the vector based on the expression of GFP in the cardiomyocytes were transferred into 96-well plates (4 larvae/well), and the β-cells were manually counted using an inverted fluorescence microscope to assess β-cell regeneration.

For cloning the *folr1* without the GPI anchor domain (named *folr1-ΔGPI*), we used the reverse primer: 5′ GGGGACCACTTTGTACAAGAAAGCTGGGTTCAGTAATACTCGGCCACTTTCC 3′ together with the forward primer used to clone *folr1* in the middle entry vector. This resulted in a 75-nucleotide deletion, i.e., a 25 amino acid deletion at the most C-terminal part of the protein (a stop codon was included in the end of the new sequence). For cloning the *tp1:folr1-NLS* construct, we amplified *folr1* from the original plasmid with primers to remove the predicted signal peptide sequence from the N-terminus and add the SV40 NLS signal (DPKKKRKV) to its C-terminus. Forward primer: 5′ GGGGACAAGTTTGTACAAAAAAGCAGGCTGCCACCATGGCTGTAGACAAGCTCAACAT 3′. Then we used two subsequent PCR reactions with two different reverse primers the first to attach the NLS signal and then add the attL2 sequence for the recombination. Reverse primer 1: 5′ CTATCACTTCTTGTATACCTTTCTCTTCTTTTTTGGATCGGAGCGCAGCAGCATCACCA 3′ and reverse primer 2: 5′ GGGGACCACTTTGTACAAGAAAGCTGGGTCTATCACTTCTTGTATACCTT 3′.

Both truncated *folr1* constructs were expressed under the control of the *tp1* promoter, and 1 nl of 15 ng/μl plasmids was injected in parallel with the full-length *tp1:folr1* construct to assess the effect on β-cell regeneration.

### Immunofluorescence, EdU incorporation, and confocal analysis

Immunofluorescence of zebrafish larvae and confocal analysis were performed as previously described. The primary antibodies used for this study were anti-GFP (1:500; Aves Labs GFP-1020), anti-insulin (1:100; custom made by Cambridge Research Biochemicals), anti-Nkx6.1 (1:50; DSHB), and anti-Folr1 (1:50; Aviva Systems Biology-ARP41427_P050).

For measuring EdU incorporation, larvae were incubated with 2 mM EdU along with 10 mM HEPES, and the signal was developed using the Click-IT EdU Alexa Fluor 647 kit (Thermo Fischer Scientific). Some of the images have the contrast setting adjusted for visualization purposes.

### Staining of mouse and human pancreata

Fixed sections of mouse pancreata were rehydrated, followed by antigen retrieval and overnight incubation with primary antibodies against GCG (1:200; G2654, Sigma-Aldrich), FOLR1 (1:100; AF6936, R&D Systems), and fluorescein-labeled DBA (1:100; FL-1031, Vector Laboratories). After washing the sections, Alexa Fluor secondary antibodies (1:200; Thermo Fisher Scientific) were incubated with the sections along with DAPI (D1306, Thermo Fisher Scientific), and then the sections were mounted and imaged.

Human tissues were kindly provided by the Alberta Diabetes Institutes Islet Core, and ethical approval for the use of human samples was obtained from the University of Alberta’s Human Research Ethics Board protocol PRO00001416. In all cases, informed written consent was provided at the institutions where the organs were harvested. Pancreatic sections from eight donors were examined as part of this study and donor information is provided in Supplementary Table 2. Antigen retrieval was used to unmask the epitopes (citrate buffer pH 5.5). Then the sections were stained with an anti-FOLR1 antibody (1:100; ARP41427_P050; Aviva System Biology), an anti-CK19 antibody (1:50; M088801-2; Agilent), and nuclei were counterstained with DAPI.

### Single-cell RNA-Seq analysis

For the single-cell RNA-Seq analysis, we used the dataset from Segerstolpe et al.^35^ with accession number E-MTAB-5061. R software was used to extract the expression data from the raw data, and Spearman’s correlation was calculated with the use of the scran package^52^. A cut-off of FDR < 0.01 was applied to test for the significance of the correlations.

### Metabolomics characterization of zebrafish larvae

Metabolites were extracted using a method adapted for zebrafish from previous work^53,54^. Four different treatment groups were used for metabolite characterization: 5 dpf larvae with/without β-cell ablation and with/without folinic acid treatment. Metabolites were extracted from five pooled larvae, and six replicates were used per condition. The zebrafish were anesthetized for collection and washed two times with cold high performance liquid chromatography-grade water. Then the zebrafish were immediately snap-frozen and lysed in 200 μl of an 80% methanol/water mixture on ice. Following homogenization, an additional 300 μl of 80% methanol/water mixture was supplemented to the tubes, followed by incubation on ice and centrifugation at 20,000*g* for 10 min. All steps were done on ice or at 4° to minimize metabolite degradation. After centrifugation, the supernatant which contains polar metabolites were evenly split into two portions and transferred into new Eppendorf tubes. After that, the methanol and water in the metabolite extract were evaporated using a speed vacuum concentrator and dried pellets were stored in a −80 °C freezer until ready for liquid chromatography–mass spectrometry (LC–MS) analysis. To re-constitute metabolites, 15 μl water was first added and vigorously vortexed for 1 min, and then 15 μl acetonitrile/methanol (1:1, v/v) was added, followed by vigorous vortexing for another 1 min. The final sample solvent is water/acetonitrile/methanol (2:1:1, v/v). Samples were then centrifuged at 20,000*g* for 5 min at 4 °C. Totally, 25 μl supernatant was transferred to an LC vial for LC–MS analysis. Some of the replicates were used to optimize the analysis of polar metabolites and folate derivatives using Vanquish UHPLC (Thermo Fisher Scientific). For this polar metabolite analysis, a hydrophilic interaction chromatography method with an Xbridge amide column (100× 2.1 mm i.d., 3.5 µm; Waters) was used for compound separation at 25 °C. Mobile phase A: water with 5 mM ammonium acetate (pH 6.8), and mobile phase B: 100% acetonitrile. Linear gradient was: 0 min, 85% B; 1.5 min, 85% B; 5.5 min, 35% B; 6.9 min, 35% B; 10.5 min, 35% B; 10.6 min, 10% B; 12.5 min, 10% B; 13.5 min, 85% B; 17.9 min, 85% B; 18 min, 85% B; 20 min, 85% B. The flow rate was: 0–5.5 min, 0.11 ml/min; 6.9–10.5 min, 0.13 ml/min; 10.6–17.9 min, 0.25 ml/min; 18–20 min, 0.11 ml/min. The analysis of folic acid and its derivatives was performed using Luna column (100× 3 mm i.d., 3 µm, C18(2), 100 Å, Phenomenex) at 25 °C. Mobile phase A: water with 0.1% formic acid, and mobile phase B: 100% methanol. Linear gradient was: 0 min, 2% B; 1.5 min, 2% B; 3.0 min, 15% B; 5.5 min, 95% B; 14.5 min, 95% B; 15.0 min, 2% B; 22.0 min, 2% B. The flow rate was 0.150 ml/min.

The mass spectrometry analysis of polar metabolites and folate derivatives was performed using Orbitrap Exploris 480 mass spectrometer (Thermo Fisher Scientific). To analyze polar metabolites, the mass spectrometer was equipped with a HESI probe and operated in the positive/negative switching mode. The relevant parameters were as listed: vaporizer temperature, 350 °C; ion transfer tube temperature, 300 °C; sheath gas, 35; auxiliary gas, 7; sweep gas, 1; spray voltage, 3.5 kV for positive mode and 2.5 kV for negative mode; RF-lens (%), 30. The resolution was set at 60,000 (at *m/z* 200). Automatic maximum injection time (max IT) and automated gain control (AGC) were used. To measure folate derivatives, Orbitrap Exploris 480 was operated in SIM mode (selected ion monitoring) targeting the following ions: folic acid (442.1470), 5-methyltetrahydrofolate (460.1939), and folinic acid (474.1732).

Differential metabolic profiles analysis was done using the Morpheus tool of the Broad institute^55^ and Metaboanalyst 4.0 was used for the pathway enrichment analysis^56^. The pathway impact value was calculated by dividing the number of significantly affected metabolites in a given pathway divided by the total number of detected metabolites in the same pathway. In the cases when only one metabolite from a given pathway was detected, and this metabolite significantly changed resulting in a pathway impact value of 1, the data are not shown as we reasoned it is hard to assess the extent the pathway actually was affected. These pathways included: linoleic acid metabolism, mannose type O-glycan biosynthesis and N-glycan biosynthesis for Fig. 6i and linoleic acid metabolism, mannose type O-glycan biosynthesis and synthesis and degradation of ketone bodies for Supplementary Fig. 6a.

### Neonatal pig islet culture and procedures

Collection of porcine pancreatic tissue and experimental procedures were conducted in accordance with the institutional ethical committee of the University of Alberta and the Canadian Council of Animal Care. Briefly, donor pancreata were surgically removed from either sex neonatal piglets (Swine Research and Technology Center, University of Alberta, Edmonton AB CAN). Isolation and culture of neonatal porcine islets were performed as characterized previously^57^. Islets were treated with 10 μM folinic for 5 days. Then, the islets were either fixed, sectioned, and stained for insulin (1:5; DAKO, code# IR002), glucagon (1:5000; Sigma-Aldrich), somatostatin (1:300; DAKO, code# A0566), and CK7 (3:100; DAKO, clone OV/TL 12/30) or further processed for GSIS analysis as previously described^58^.

### Statistical analysis

Statistical analysis was carried out using GraphPad Prism software. *P* values $\le$ 0.05 were considered statistically significant. The normality of the data was estimated using the Shapiro-Wilk normality test. Representative data are shown in each figure unless the data were pooled together from the independent experiments, in which case this method is mentioned in the figure legend. The statistical tests and exact *P* values are stated in the figure legends for each experiment.

### Reporting Summary

Further information on research design is available in the Nature Research Reporting Summary linked to this article.

## Supplementary information

Supplementary information

Description of Additional Supplementary Files

Supplementary Data 1

Reporting Summary

## Data Availability

All relevant data and reagents are available upon request to the corresponding author O.A. The raw data of the metabolomics study are deposited in Metabolomics Workbench^59^ with the study ID ST001670 [10.21228/M80Q4N]. The raw reads of the RNA-Seq study are publicly accessible in the Sequence Read Archive with the project ID SRP315319. Source data are provided with this paper.

## Source data

Source data
