# Supplementary material for: Reinforcing one-carbon metabolism via folic acid/Folr1 promotes β-cell differentiation
Source: Nat Commun. 2021 Jun 7;12:3362. doi: 10.1038/s41467-021-23673-0 (PMC8184927; doi:10.1038/s41467-021-23673-0)
Supplement: Supplementary file 1 — Supplementary information [file 41467_2021_23673_MOESM1_ESM.pdf]

Supplementary information

Reinforcing one-carbon metabolism via folic acid/Folr1 promotes β-cell differentiation

Christos Karampelias[1], Habib Rezanejad[2], Mandy Rosko[2], Likun Duan[3], Jing Lu[1], Laura Pazzagli[4], Philippe Bertolino[5], Carolyn E. Cesta[4], Xiaojing Liu[3], Gregory S. Korbutt[2], Olov Andersson[1*]

[1]Department of Cell and Molecular Biology, Karolinska Institutet, Stockholm, Sweden

[2]Alberta Diabetes Institute, University of Alberta, Edmonton, Alberta, Canada

[3]Department of Molecular and Structure Biochemistry, NC State University, Raleigh, NC 27695, USA

[4]Centre for Pharmacoepidemiology, Department of Medicine Solna, Karolinska Institutet, Stockholm, Sweden

[5]Cancer Research Centre of Lyon, INSERM U1052, CNRS UMR5286, Claude Bernard University, Lyon, France

[*]Correspondence. Email: olov.andersson@ki.se; Phone: +46(0)733462929

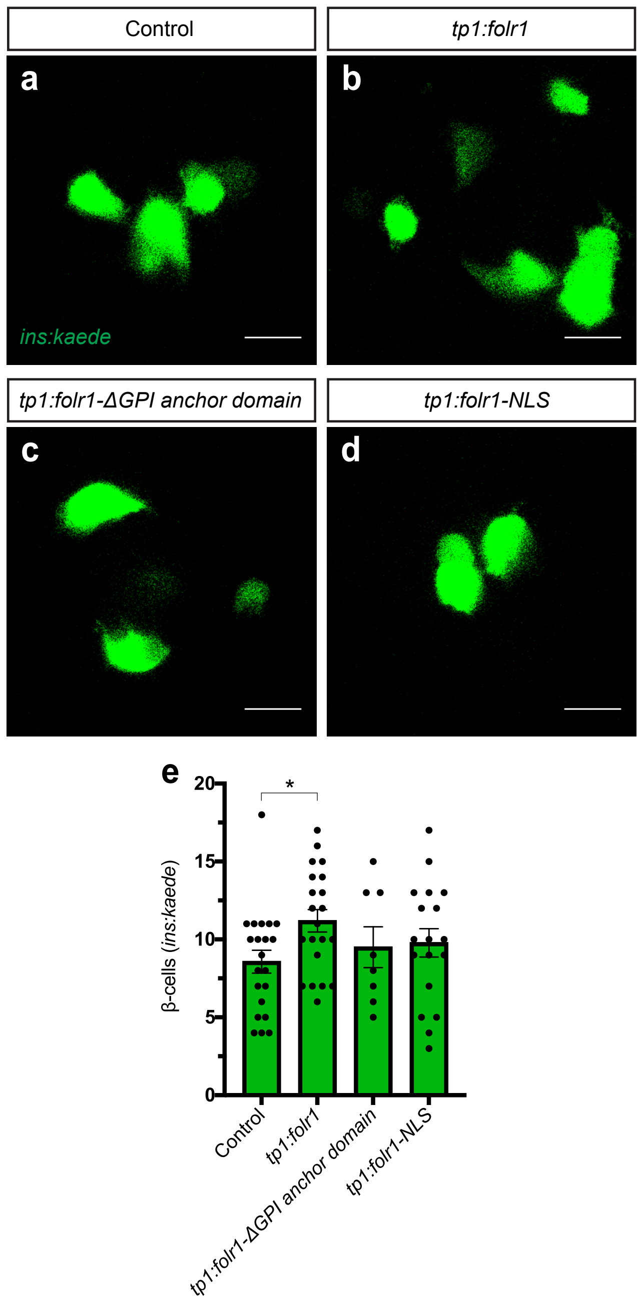

Supplementary Figure 1: Overexpression of a *folr1* lacking the GPI anchor domain does not increase β-cell regeneration.

a-e, Single-plane confocal images of islets in control (**a**), *tp1:folr1* (**b**), *tp1:folr1-ΔGPI* anchor domain (**c**) and *tp1:folr1-NLS* (**d**) overexpressing larvae in *Tg(ins:kaede;ins:CFP-NTR)*, following two days of β-cell regeneration. Quantification (**e**) showed a significant increase in β-cell regeneration with the *tp1:folr1* but not the *tp1:folr1-ΔGPI* anchor domain or the *tp1:folr1-NLS* construct. Scale bar, 10 μm. *n*=21

(control), *n*=21 (*tp1:folr1*), *n*=8 (*tp1:folr1-ΔGPI* anchor domain) and *n*=18 (*tp1:folr1-NLS*) biologically independent zebrafish larvae were used for the quantification of β-cells. Data are presented as mean values ±SEM. One-way ANOVA followed by a Sidák's multiple comparison test was used to assess significance. (**e**) **P*=0.0376.

a

**Gene names**	**rho**
CFH	0,274
TSPAN1	0,272
CEACAM6	0,272
SLPI	0,251
EDN2	0,239
CCL28	0,239
B4GALT4	0,235
ABCB1	0,228
SYT8	0,224
MFGE8	0,223
MISP	0,219
VNN2	0,218
MUC1	0,217
SORL1	0,214
CYP3A5	0,213
APCDD1	0,212
TIMP1	0,210
EFNB1	0,209
IGFBP3	0,208
WDR72	0,208
B3GNT3	0,207
TNIK	0,206
MMP24-AS1	0,206
FOS	0,205
GPR110	0,205
TRO	0,204
SLC9A3R2	0,202
CAPN5	0,202

Supplementary Figure 2: Genes correlating with *FOLR1* expression in the human ductal cells.

a, Table showing the genes that positively correlated with *FOLR1* expression in the ductal cells of the single-cell RNA-Seq dataset (Fig. 3**d**-**f**).

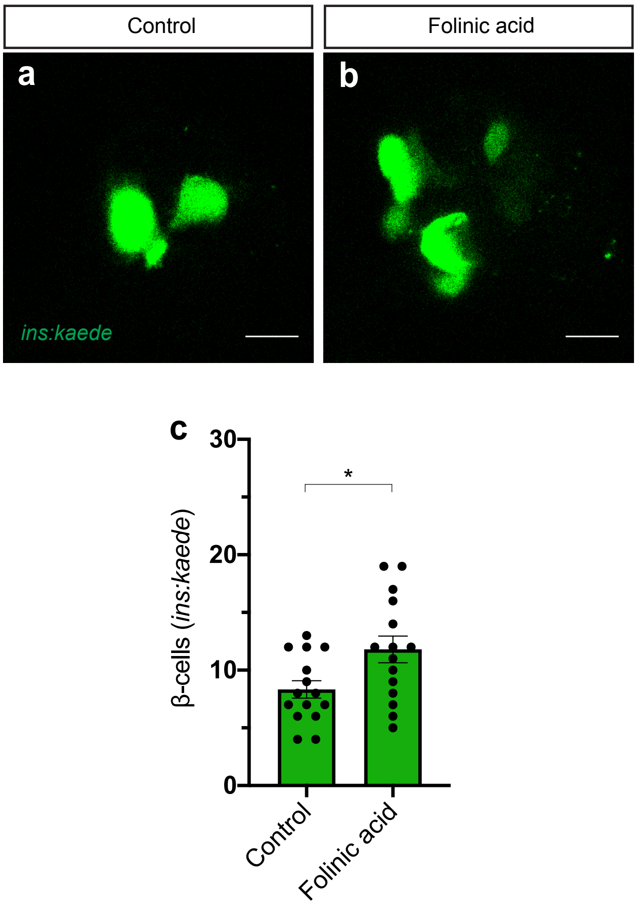

Supplementary Figure 3: Folinic acid treatment increase β-cell regeneration.

a-c, Single-plane confocal images of islets in control (**a**) or folinic acid-treated (**b**) *Tg(ins:kaede;ins:CFP-NTR)* zebrafish larvae following two days of β-cell regeneration. Quantification (**c**) showed a significant increase in β-cell regeneration. Scale bar, 10 μm. *n*=15 biologically independent zebrafish larvae were used for the quantification of β-cells in both the control and folinic acid-treated larvae. Data are presented as mean values ±SEM. Unpaired two-tailed Student's *t*-test was used to assess significance. (**c**) **P*=0.0183.

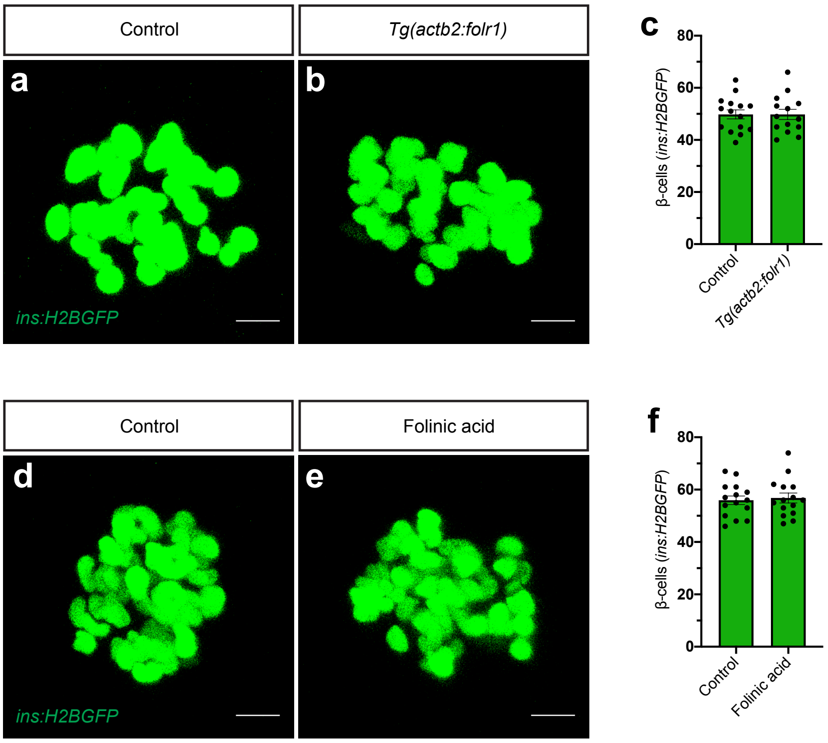

Supplementary Figure 4: One-carbon metabolism reinforcement does not alter the β-cell number in the basal state.

a-**c**, Single-plane confocal images of islets in control (**a**) and *Tg(actb2:folr1)* (**b**) zebrafish larvae at 6 dpf. *Tg(ins:H2BGFP)* was used to visualize the β-cells. Quantification of the number of β-cells (**c**) showed no change upon *folr1* overexpression in the basal state. Scale bar, 10 μm. *n*=15 control, and *n*=14 *Tg(actb2:folr1)* biologically independent zebrafish larvae were used for the quantification of β-cells. Data are presented as mean values ±SEM.

d-**f**, Single-plane confocal images of islets in 6 dpf zebrafish larvae after two days of control (**d**) or folinic acid treatment (**e**). Similar to the *Tg(actb2:folr1)* line, folinic acid treatment did not affect the number of β-cells in the basal state, as quantified in (**f**). Scale bar, 10 μm. *n*=15 biologically independent zebrafish larvae were used for the quantification of β-cells in both the control and folinic acid-treated larvae. Data are presented as mean values ±SEM.

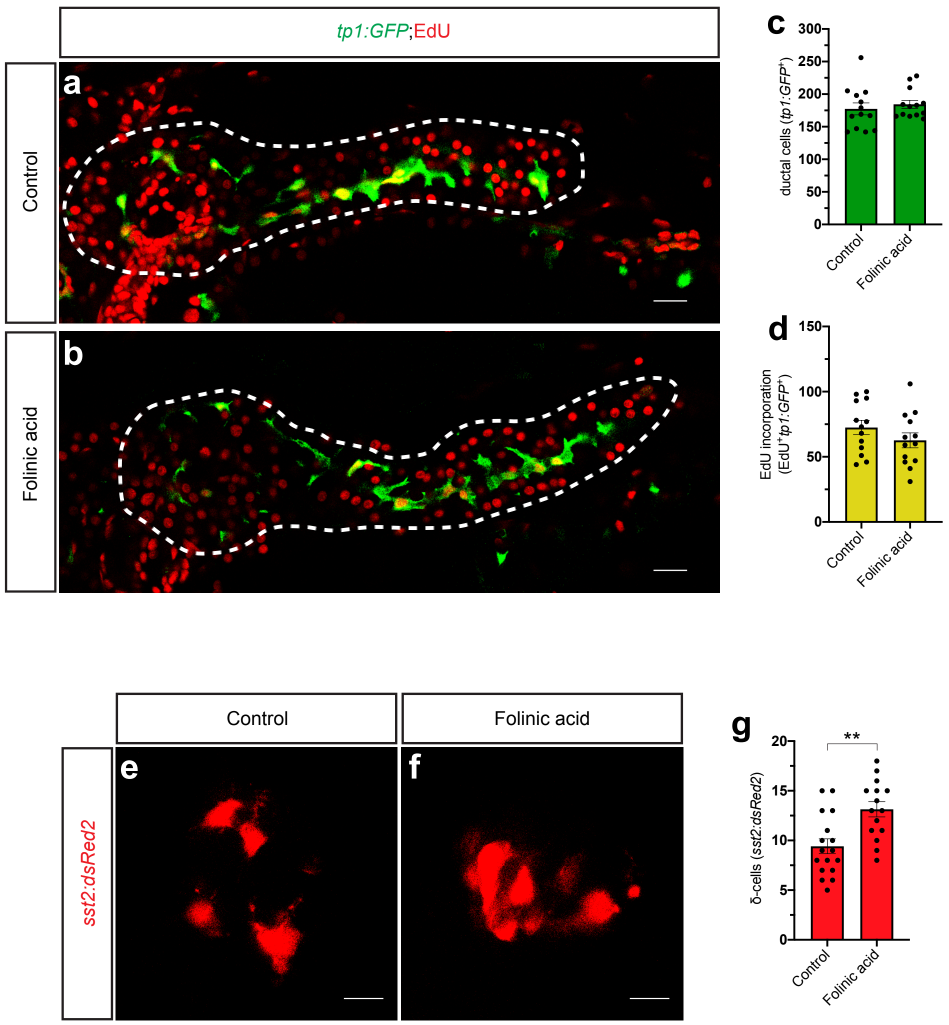

Supplementary Figure 5: Folinic acid does not affect the number of ductal cells but stimulates δ-cell regeneration in zebrafish.

a-**d**, Single-plane confocal images of pancreata in control (**a**) or folinic acid-treated (**b**) larvae in the *Tg(tp1:GFP)*;*Tg(ins:flag-NTR)* background for visualization of ductal cells. EdU (red) marks proliferating cells. The white dashed line outlines the pancreas. Quantification revealed no change in the number of *tp1:GFP*$^{+}$ cells (**c**) or their proliferation status (**d**). Scale bar, 20 μm. *n*=13 biologically independent zebrafish larvae were used for the quantification of both the control and folinic acid-treated larvae. Data are presented as mean values ±SEM.

e-**g**, Single-plane confocal images of islets in 6 dpf *Tg(sst2:NTR)*;*Tg(sst2:dsRed2)* larvae after δ-cell ablation and two days of control (**e**) or folinic acid treatment (**f**).

Quantification showed that folinic acid treatment stimulated δ-cell regeneration (**g**). Scale bar, 10 μm. *n*=17 (control) and *n*=15 (folinic acid) biologically independent zebrafish larvae were used for the quantification of δ-cells; Data are presented as mean values ±SEM. Unpaired two-tailed Student's *t*-test was used to assess significance. (**g**) $^{**}P=0.0016$.

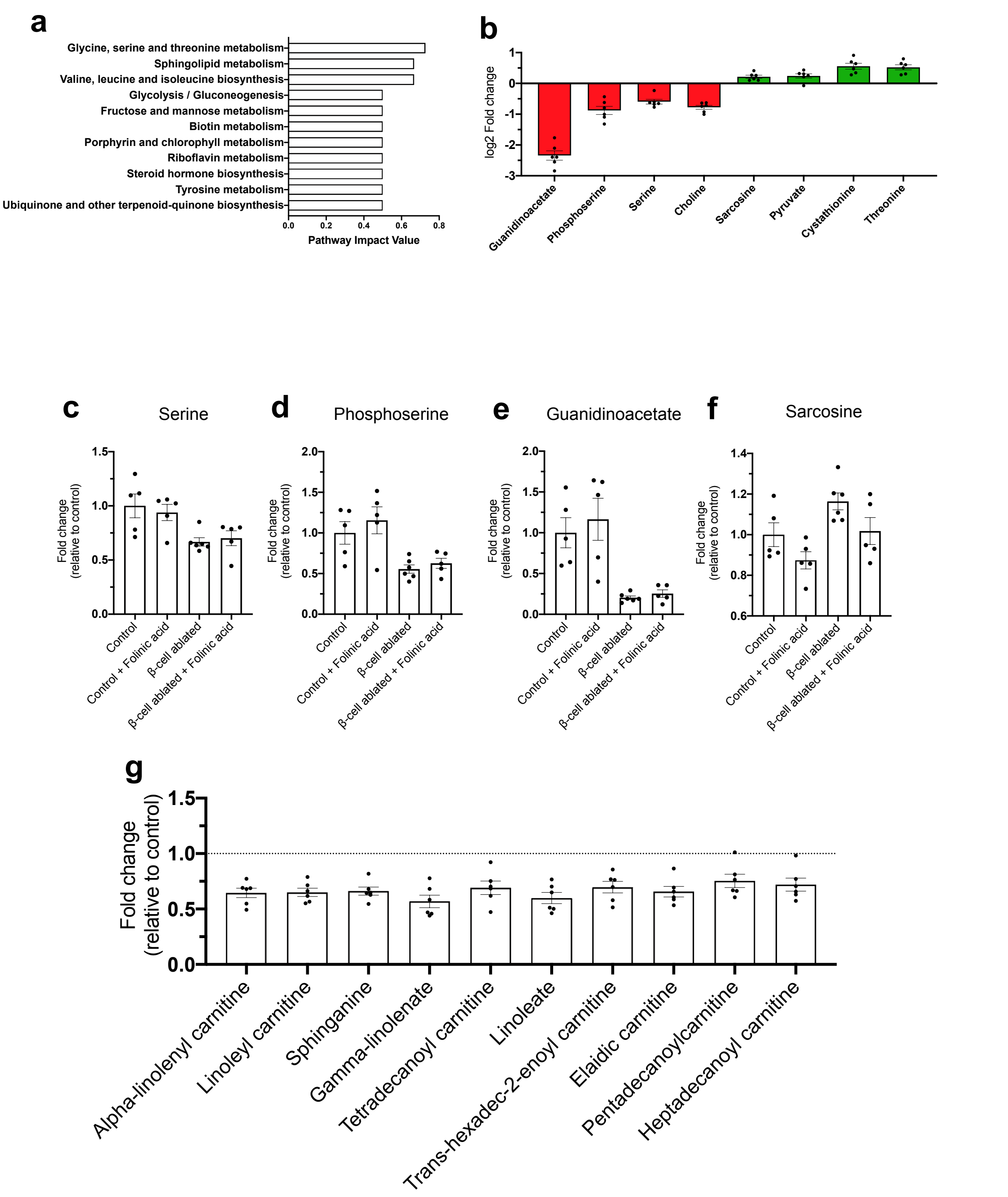

Supplementary Figure 6: Metabolomics characterization of the basal and β-cell regeneration state in zebrafish larvae.

a, Pathway impact value of zebrafish-specific pathway analysis for metabolites with significantly altered levels following β-cell ablation.

b, log_2 fold change of the significantly changed metabolites of the glycine, serine and threonine pathway, i.e. in larvae following β-cell ablation compared to larvae in the

basal state. *n*=5 (control) and *n*=6 (β-cell ablated) biologically independent metabolite preparations from 5 pooled larvae each were used for this experiment.

c-**f**, Fold change of the level of serine (**c**), phosphoserine (**d**), guanidinoacetate (**e**) and sarcosine (**f**) between all four different conditions in comparison to the control (basal state). *n*=5 (control), *n*=5 (control + folinic acid), *n*=6 (β-cell ablated) and *n*=5 (β-cell ablated + folinic acid) biologically independent metabolite preparations from 5 pooled larvae each were used for the quantification of this experiment.

g, Fold change of certain significantly decreased metabolites following β-cell ablation, levels that are partially reversed upon folinic acid treatment (Fig. 6b). *n*=5 (control) and *n*=6 (β-cell ablated) biologically independent metabolite preparations from 5 pooled larvae each were used for this experiment.

All data in this figure are shown as mean values ±SEM.

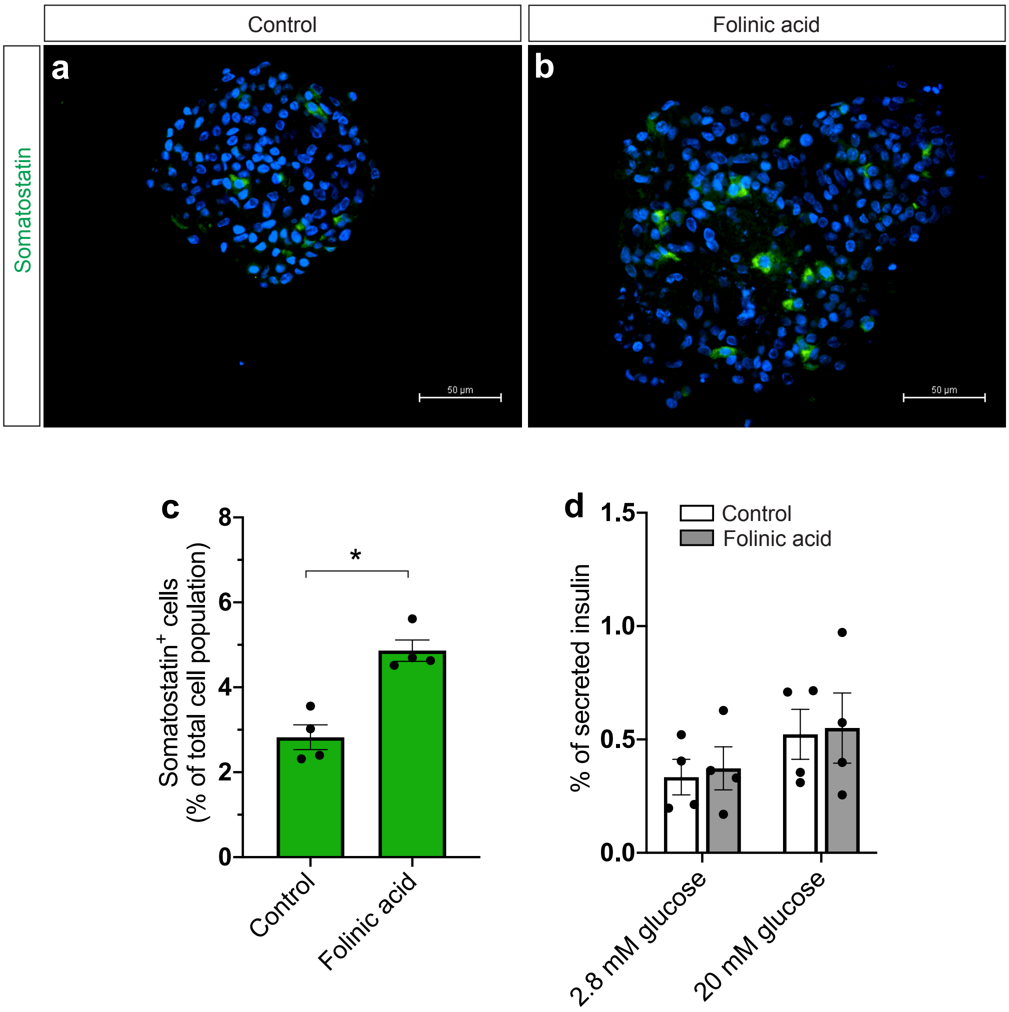

Supplementary Figure 7: Folinic acid increases δ-cell differentiation in neonatal pig islets.

a-c: Folinic acid treatment stimulate the differentiation of δ-cells in neonatal pig islets. Images of control (**a**) or folinic acid-treated (**b**) neonatal pig islets immunostained for somatostatin and nuclei counterstained with DAPI. Quantification of the percentage of SST^+ cells showed an increase after folinic acid treatment (**c**). Scale bar, 50 µm. *n*=4 biologically independent pig islet preparations; Data are presented as mean values ±SEM. Mann-Whitney two-tailed test was used to calculate the statistical significance. (**c**) **P* =0.0286.

d, Glucose stimulated insulin secretion of control or folinic acid-treated pig islets. Values are shown as mean ± SEM. *n*=4 biologically independent pig islet preparations.

Supplementary table 1: Primers used to amplify the genes of interest for cloning into the middle donor vector of the Gateway system.

Gene	Forward primer	Reverse primer
adma	5’-GGGGACAAGTTTGTACAAAAAAGCAGGCTGCCACCATGCAACTGATCCTGCAGTC-3’	5’-GGGGACCACTTTGTACAAGAAAGCTGGGTTCACGTCCGTCTGAGCAGCG-3’
agt	5’-GGGGACAAGTTTGTACAAAAAAGCAGGCTGCCACCATGAAGATGTTCCTCGCTTT-3’	5’-GGGGACCACTTTGTACAAGAAAGCTGGGTTTAGGCTGTTGGGTTGATGA-3’
apoa1a	5’-GGGGACAAGTTTGTACAAAAAAGCAGGCTGCCACCATGAAATTCGTGGCTCTTGC-3’	5’-GGGGACCACTTTGTACAAGAAAGCTGGGTTTATGCCTGGATGGCCTTGG-3’
bgnb	5’-GGGGACAAGTTTGTACAAAAAAGCAGGCTGCCACCATGTCAGTGATGTTCTCCTG-3’	5’-GGGGACCACTTTGTACAAGAAAGCTGGGTCTATTTTTTATAGTTTCCAA-3’

ephrina2	5'-GGGGACAAGTTTGTACAAAAAAGCAGGCTGCCACCATGGACAAGAACTTTACTAT-3'	5'-GGGGACCACTTTGTACAAGAAAGCTGGGTCTATGAGGAACACAGATGTA-3'
folr1	5'-GGGGACAAGTTTGTACAAAAAAGCAGGCTGCCACCATGGACGCTTTATTTCACAG-3'	5'-GGGGACCACTTTGTACAAGAAAGCTGGGTTCAGCGCAGCAGCATCACCA-3'
galn	5'-GGGGACAAGTTTGTACAAAAAAGCAGGCTGCCACCATGCACAGGTGTGTCGGTGG-3'	5'-GGGGACCACTTTGTACAAGAAAGCTGGGTTTAGGGTTGACTGATCTCTT-3'
ier3ip1	5'-GGGGACAAGTTTGTACAAAAAAGCAGGCTGCCACCATGGCGTTCACACTGTACGC-3'	5'-GGGGACCACTTTGTACAAGAAAGCTGGGTTCATCCAAACAACAAAAGTA-3'
penka	5'-GGGGACAAGTTTGTACAAAAAAGCAGGCTGCCACCA	5'-GGGGACCACTTTGTACAAGAAAGCTGGGTCTAATCCATGAATCCTCCGT-3'

	TGGCGTTAATGATGAACTC -3’	
serpina7	5’- GGGGACAAGTTTGTACAA AAAAGCAGGCTGCCACCA TGGAACAGAACGGTGTAT T-3’	5’- GGGGACCACTTTGTACAAGA AAGCTGGGTTCACAGTTTTT CATTTGGGT-3’
sfrp5	5’- GGGGACAAGTTTGTACAA AAAAGCAGGCTGCCACCA TGGCGGAGCTGAAGAGGC A-3’	5’- GGGGACCACTTTGTACAAGA AAGCTGGGTTCACTGGAAGA CACTGTGAT-3’
sostdc1a	5’- GGGGACAAGTTTGTACAA AAAAGCAGGCTGCCACCA TGTATATAAACGCACCAGA -3’	5’- GGGGACCACTTTGTACAAGA AAGCTGGGTCTATATTAAGG TCAGTTCAA-3’
spint1b	5’- GGGGACAAGTTTGTACAA AAAAGCAGGCTGCCACCA TGGGTTCGTGGTGTTTTCT -3’	5’- GGGGACCACTTTGTACAAGA AAGCTGGGTTCATGCTTTGG CAGTGGTGC-3’
zgc:163030	5’- GGGGACAAGTTTGTACAA AAAAGCAGGCTGCCACCA	5’- GGGGACCACTTTGTACAAGA

	TGTTTCTTAAAGCTATTGT-3’	AAGCTGGGTCTACAGGCAGGAATTTTCAG-3’
zgc:174259	5’-GGGGACAAGTTTGTACAAAAAAGCAGGCTGCCACCATGAACAACGATTTTGCTTT-3’	5’-GGGGACCACTTTGTACAAGAAAGCTGGGTTCATAGTTTTTCATTTGGGT-3’
zgc:198329	5’-GGGGACAAGTTTGTACAAAAAAGCAGGCTGCCACCATGATGGTTTCGCATGGTTT-3’	5’-GGGGACCACTTTGTACAAGAAAGCTGGGTTCACCTTTGACTGCTTGGAA-3’

Supplementary table 2: Relevant population characteristics of the human donors (3 males and 5 females) whose pancreatic tissue was used for assessing FOLR1 expression.

Donor Code	Age	BMI	Cold Ischemia Time (h)
R005	1.58	19.8	5
R033	10	16.7	Not Available
R269	14	21.5	16
R045	27	19.4	5
R029	33	27.3	12
R044	38	24.6	15
R271	60	25.9	13.5
R200	65	27.1	17.5
