# Supplementary material for: Reinforcing one-carbon metabolism via folic acid/Folr1 promotes β-cell differentiation
Source: Nat Commun. 2021 Jun 7;12:3362. doi: 10.1038/s41467-021-23673-0 (PMC8184927; doi:10.1038/s41467-021-23673-0)
Supplement: Supplementary file 2 — Description of Additional Supplementary Files [file 41467_2021_23673_MOESM2_ESM.docx]

Description of Additional Supplementary Files

Title: Supplementary Data 1

Description: List of zebrafish metabolites that are significantly altered by β-cell ablation, along with their respective fold change.
