# Supplementary material for: Reinforcing one-carbon metabolism via folic acid/Folr1 promotes β-cell differentiation
Source: Nat Commun. 2021 Jun 7;12:3362. doi: 10.1038/s41467-021-23673-0 (PMC8184927; doi:10.1038/s41467-021-23673-0)
Supplement: Supplementary file 4 — Reporting Summary [file 41467_2021_23673_MOESM4_ESM.pdf]

nature research

Corresponding author(s): Olov Andersson

Last updated by author(s): Apr 21, 2021

Reporting Summary

Nature Research wishes to improve the reproducibility of the work that we publish. This form provides structure for consistency and transparency in reporting. For further information on Nature Research policies, see our Editorial Policies and the Editorial Policy Checklist.

Statistics

For all statistical analyses, confirm that the following items are present in the figure legend, table legend, main text, or Methods section.

n/a	Confirmed	
☐	☒	The exact sample size (*n*) for each experimental group/condition, given as a discrete number and unit of measurement
☐	☒	A statement on whether measurements were taken from distinct samples or whether the same sample was measured repeatedly
☐	☒	The statistical test(s) used AND whether they are one- or two-sided<br>*Only common tests should be described solely by name; describe more complex techniques in the Methods section.*
☒	☐	A description of all covariates tested
☐	☒	A description of any assumptions or corrections, such as tests of normality and adjustment for multiple comparisons
☐	☒	A full description of the statistical parameters including central tendency (e.g. means) or other basic estimates (e.g. regression coefficient) AND variation (e.g. standard deviation) or associated estimates of uncertainty (e.g. confidence intervals)
☐	☒	For null hypothesis testing, the test statistic (e.g. *F*, *t*, *r*) with confidence intervals, effect sizes, degrees of freedom and *P* value noted<br>*Give P values as exact values whenever suitable.*
☒	☐	For Bayesian analysis, information on the choice of priors and Markov chain Monte Carlo settings
☒	☐	For hierarchical and complex designs, identification of the appropriate level for tests and full reporting of outcomes
☐	☒	Estimates of effect sizes (e.g. Cohen's *d*, Pearson's *r*), indicating how they were calculated

Our web collection on statistics for biologists contains articles on many of the points above.

Software and code

Policy information about availability of computer code

Data collection	LAS X v3.5.5.19976 (for confocal microscopy-v3.5.5.19976), NIS-Elements 4.30 (for pig islet and human stainings image acquisition) and Xcalibur 4.4 (for mass spectrometer data collection).
Data analysis	Fiji/ImageJ (for image analysis - version 2.0.0-rc-65/1.51w), Excel (version 16.16.27), GraphPad PRISM 8.0, RStudio, R version 3.3.3 and R package scran. Bowtie2 v2.2.2 (filtering) and TopHat 2.0.10 (alignment). SignalP v4.0 was used to call potentially secreted proteins. Sieve 2.2 was used for chromatographic alignment and peak integration. Morpheus tool from Broad Institute and Metaboanalyst 4.0 was used to analyze the metabolomics data.

For manuscripts utilizing custom algorithms or software that are central to the research but not yet described in published literature, software must be made available to editors and reviewers. We strongly encourage code deposition in a community repository (e.g. GitHub). See the Nature Research guidelines for submitting code & software for further information.

Data

Policy information about availability of data

All manuscripts must include a data availability statement. This statement should provide the following information, where applicable:
- Accession codes, unique identifiers, or web links for publicly available datasets
- A list of figures that have associated raw data
- A description of any restrictions on data availability

All relevant data and reagents are available upon request to the corresponding author O.A. The raw data of the metabolomics study are deposited in Metabolomics Workbench59 with the study ID ST001670 [https://doi.org/10.21228/M80Q4N]. The raw reads of the RNA-Seq study are publicly accessible in the Sequence Read Archive with the project ID SRP315319 [https://www.ncbi.nlm.nih.gov/sra/?term=SRP315319]. Source data are provided with this paper.

Field-specific reporting

Please select the one below that is the best fit for your research. If you are not sure, read the appropriate sections before making your selection.

[x] Life sciences [ ] Behavioural & social sciences [ ] Ecological, evolutionary & environmental sciences

For a reference copy of the document with all sections, see nature.com/documents/nr-reporting-summary-flat.pdf

Life sciences study design

All studies must disclose on these points even when the disclosure is negative.

Sample size	The sample number was not predetermined for any of the experiments. We considered a sufficient final sample size when a statistical significant value for group comparison was reached.<br>Experiments regarding in vivo zebrafish work as well as in vitro work with pig islets have been repeated at least twice. Each biological replicate contained at least 4 larvae/adult zebrafish or pig islet preparations.
Data exclusions	No data have been excluded from the final analysis.
Replication	All experiments have been reproduced at least two times, with the majority of them at least three times. All experiments were reproducible.
Randomization	All experiments including larvae and juvenile zebrafish, pig islet preparations, mouse and human sections were randomly assigned to each experimental condition.
Blinding	Investigators were not blinded for any of the experiments reported. When the experimental design required to allocation to specific groups or treatments no blinding was required. When different treatment groups were involved the data collection was unbiased and the different groups were blinded for the analysis of these datasets.

Reporting for specific materials, systems and methods

We require information from authors about some types of materials, experimental systems and methods used in many studies. Here, indicate whether each material, system or method listed is relevant to your study. If you are not sure if a list item applies to your research, read the appropriate section before selecting a response.

Materials & experimental systems

n/a	Involved in the study	
[ ]	[x]	Antibodies
[x]	[ ]	Eukaryotic cell lines
[x]	[ ]	Palaeontology and archaeology
[ ]	[x]	Animals and other organisms
[ ]	[x]	Human research participants
[x]	[ ]	Clinical data
[x]	[ ]	Dual use research of concern

Methods

n/a	Involved in the study	
[x]	[ ]	ChIP-seq
[x]	[ ]	Flow cytometry
[x]	[ ]	MRI-based neuroimaging

Antibodies

Antibodies used	For zebrafish stainings: #1 chicken anti-GFP (1:500, Aves Labs - GFP-1020), #2 rabbit anti-insulin (1:100, custom made by Cambridge Research Biochemicals), #3 mouse anti-Nkx6.1 (1:50, DSHB-F55A10).<br>Secondary antibodies used for zebrafish stainings: #1 goat anti-Chicken Alexa Fluor 488 (1:500, A-11039, ThermoFischer Scientific), #2 goat anti-Rabbit Alexa Fluor 488 (1:500, A-11034, ThermoFischer Scientific), #3 donkey anti-Mouse Alexa Fluor 546 (1:500, A10036, ThermoFischer Scientific), #4 donkey anti-Rabbit Alexa Fluor 647 (1:500, A-31573, ThermoFischer Scientific)<br><br>For mouse stainings: #1 mouse anti-glucagon (1:200, G2654, Sigma-Aldrich), #2 sheep anti-mFolr1 (1:100, AF6936, R&D Systems), #3 fluorescein-labeled DBA (1:100, FL-1031, Vector Laboratories).<br><br>For human and zebrafish stainings: rabbit anti-FOLR1 (1:100 for human sections and 1:50 for zebrafish whole-mount immunofluorescence, ARP41427_P050, Aviva System Biology). For human staining: mouse anti-CK19 (1:50, M088801-2, Clone RCK108, Agilent), goat anti-rabbit Alexa Fluor 488 (1:200, A32731, ThermoFischer Scientific) and goat anti-mouse Alexa Fluor 594 (1:200, A-11005, ThermoFischer Scientific).<br><br>For pig islet stainings: #1 rabbit anti-somatostatin (1:300, DAKO, code# A0566), #2 guinea-pig anti-insulin (1:5; DAKO, code# IR002), #3 glucagon (1:5000, Sigma-Aldrich), #4 mouse anti-CK7 (3:100, DAKO, clone OV/TL 12/30), #
Validation	For the zebrafish stainings: antibody #1 has been cited 1055 times in the literature (e.g https://doi.org/10.15252/embj.201592903),

antibody #2 has been validated using transgenic lines fluorescently labelling the beta-cells in zebrafish and antibody #3 has been previously shown to recapitulate the expression of a nkx6.1:GFP zebrafish transgenic line (Ghaye et al;BMC Biology;2015).

For mouse stainings: Antibody #1 has been cited 255 times in the literature (e.g mouse pancreata staining: doi: 10.1111/j.1469-7580.2004.00265.x). Antibody #3 has been cited 17 times (e.g mouse pancreata staining:DOI: 10.1016/j.celrep.2016.03.036). The folr1 antibody has been used in a previous mouse study (Gennet et al;Scientific reports;2016) and has been validated by the supplier.

For the human and zebrafish stainings the Folr1 antibody has been validated by the supplier with the the use of a blocking peptide in human pancreatic cells (see here: https://www.avivasysbio.com/folr1-antibody-middle-region-arp41427-p050.html). The mouse anti-CK19 antibody has been cited 91 times (e.g. DOI: 10.1038/s41598-019-40481-1)

For antibodies used in the pig islet stainings, the antibodies were used in previous studies (Hassouna et al; Transplantation; 2018)

Animals and other organisms

Policy information about studies involving animals; ARRIVE guidelines recommended for reporting animal research

Laboratory animals	For zebrafish experiments either the AB or the TL background strain was used. The transgenic lines used were: Tg(ins:flag-NTR), Tg (tp1:H2BmCherry), Tg(tp1:GFP), Tg(ins:CFP-NTR), Tg(ins:Kaede), Tg(ins:H2BGFP), Tg(sst2:NTR,cryaa:Cerulean), Tg(sst2:dsRed2) and Tg (actb2:folr1)<br>Two ages of zebrafish were used for the experiments: Larvae (up to 6 dpf) and juvenile (1 month old). Sex is not possible to be determined at the larval and juvenile stages.<br>Mouse pancreatic sections were previously prepared from 10-weeks old wild-type C57Bl/6J male mice.<br>Wild type neonatal pigs of either sex were sacrificed to obtain the islet cultures for experiments.
Wild animals	The study did not involve wild animals.
Field-collected samples	The study did not involve samples collected from the field.
Ethics oversight	Work with zebrafish was approved and performed following the guidelines of Stockholms djurförsöksetiska nämnd under an approved ethical permit.<br>Work with neonatal pig islets was performed under the guidelines from Canadian Council on Animal Care.

Note that full information on the approval of the study protocol must also be provided in the manuscript.

Human research participants

Policy information about studies involving human research participants

Population characteristics	The information about the human donors in this study is shown in the supplementary table 2 of the manuscript.
Recruitment	Human tissues were kindly provided by the organ donors to the Alberta Diabetes Institutes Islet Core.
Ethics oversight	Ethical approval for the use of human samples was obtained from the University of Alberta's Human Research Ethics Board protocol PRO00001416.

Note that full information on the approval of the study protocol must also be provided in the manuscript.
